# Troglitazone, a PPAR-γ activator prevents endothelial cell adhesion molecule expression and lymphocyte adhesion mediated by TNF-α

**DOI:** 10.1186/1472-6793-5-3

**Published:** 2005-02-06

**Authors:** Makoto Sasaki, Paul Jordan, Tomas Welbourne, Alireza Minagar, Takashi Joh, Makoto Itoh, John W Elrod, J Steven Alexander

**Affiliations:** 1 Department of Molecular and Cellular Physiology, LSU Health Sciences Center, Shreveport, LA, 71130, USA; 2 Department of Gastroenterology, LSU Health Sciences Center, Shreveport, LA, 71130, USA; 3 Department of Neurology, LSU Health Sciences Center, Shreveport, LA, USA, 71130, USA; 4 Nagoya City University Graduate School of Medical Sciences, Departments of Internal Medicine and Bioregulation, Nagoya City University Graduate School of Medical Sciences, 1-Kawasumi-Mizuho, Nagoya 467-8601, Japan

## Abstract

**Background:**

Cytokine mediated induction of the mucosal addressin cell adhesion molecule-1(MAdCAM-1) expression is associated with the onset and progression of inflammatory bowel disease (*IBD*).

**Results:**

Using western blotting and cell-based ELISA, we show in this study that troglitazone, an activator of the peroxisome proliferator-activated receptor-γ (PPAR-γ), widely used in the treatment of diabetes, has as well recently been highlighted as protective in models of inflammation and cancer. We found that troglitazone (10–40 μM), significantly reduced the TNF-α (1 ng/ml) mediated induction of endothelial MAdCAM-1 in a dose-dependent manner, achieving a 34.7% to 98.4% reduction in induced MAdCAM-1. Trogliazone (20μM) reduced TNF-α induced VCAM-1, ICAM-1 and E-selectin expression. Moreover, troglitazone significantly reduced α4β7-integrin dependent lymphocyte adhesion to TNF-α cultured endothelial cells.

**Conclusions:**

These results suggest that PPAR-γ agonists like troglitazone may be useful in the clinical treatment of IBD.

## Background

Endothelial cell adhesion molecules or 'ECAMs' play essential roles in the development of chronic inflammation by recruiting leukocytes, especially lymphocytes through their ability to promote leukocyte rolling, firm adhesion and extravasation [15]. Tissue infiltration by leukocytes is a common hallmark in several chronic inflammatory states which include the inflammatory bowel diseases, ulcerative colitis (UC), and Crohn's disease (CD), but also several other chronic inflammatory states such as arthritis, lupus, diabetes [17,47,58]. In the setting of IBD, the expression of ECAMs like ICAM-1, VCAM-1, and MAdCAM-1 is observed in experimental models of colitis, [11,33,34,48] and also within the inflamed human colon in Crohn's disease and ulcerative colitis [3,49]. Among the adhesion molecules that are up-regulated in IBD, MAdCAM-1, the mucosal cell adhesion molecule is thought to be preeminent in the development of chronic gut inflammation. MAdCAM-1 is normally expressed in the gut, and its expression is dramatically increased during inflammation [11,48]. The functional significance of increased expression of MAdCAM-1 in IBD is supported by several reports which demonstrate that immunoneutralization of either MAdCAM-1 or its lymphocyte ligand, the α4β7 integrin, attenuate inflammation and mucosal damage in a variety of animal models of colitis [14,24,55]. However, since monoclonal antibodies directed against other ECAMs, particularly VCAM-1, can as well reduce disease activity in animal models of colitis [2,16,46,53], the literature suggests that MAdCAM-1 is probably necessary, but insufficient for the maximal penetrance of experimental and clinical IBD. Based on these results, it is apparent that an improved understanding of the mechanisms regulating ECAM expression, especially MAdCAM-1, might help to design improved therapies for colitis.

Peroxisome proliferator-activated receptors (PPARs) are members of the nuclear hormone receptor superfamily of transcription factors, whose activities are regulated through the high affinity binding of small lipophilic ligands that include steroid hormones [29]. A new class of antidiabetic drugs, known as 'glitazones' which includes troglitazone, rosiglitazone, and pioglitazone, have been developed as agonists that bind to the gamma (γ)-subtype of the PPARs. While glitazones have been extensively used in the treatment of diabetes, several investigators have now demonstrated that PPAR-γ ligands will markedly reduce colonic inflammation of in two different mouse models of colitis [12,51]. In addition, glitazones provide some benefit in the treatment of ulcerative colitis in humans as well [27].

Although PPAR-γ is expressed at high levels in adipose tissues, PPAR-γ has also been described in many other kinds of cells, including those in the vasculature like endothelial cells, vascular smooth muscle cells and monocytes and macrophages [19]. Although it not yet completely clear, the literature suggests that glitazones may be therapeutic in these models through the ability of these PPAR-γ activators to inhibit several events in inflammation particularly leukocyte infiltration into tissues mediated by NF-kB-dependent ECAM expression [6,21,32,38,51].

However, the literature does not uniformly support protective roles for all PPARs. For example, it has been suggested that activation of *PPAR*-α, rather than PPAR-γ activation is responsible for blocking cytokine induced ECAM expression [30,41] and these differences may reflect tissue- and/or species specific responses to glitazones. Regardless, glitazones might be therapeutic in the setting of IBD through their ability to restrict expression of *MAdCAM-1*, one of the more important regulators of gut inflammation in IBD. However, this has not yet been investigated.

In the present study we have examined the ability of a candidate glitazone PPAR-γ ligand, *troglitazone*, to limit cytokine induction of MAdCAM-1 and also VCAM-1, ICAM-1 and E-selectin, and decrease MAdCAM-1 dependent lymphocyte endothelial adhesion *in vitro*.

## Results

### PPAR-γ expression by endothelial cells

**Figure 1:**
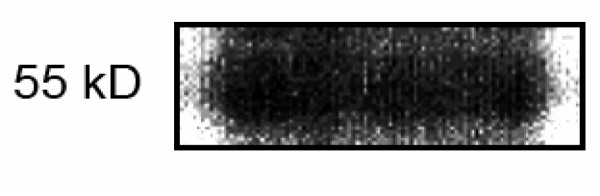
**PPAR-γ expression in SVEC endothelial cell**. Western blotting for PPAR-γ in SVEC cells reveals a 55 kD band after reacting with anti-PPAR-γ peptide antibody, indicating that PPAR-γ is in fact present within the cells used in this study.

To confirm the presence of PPAR-γ on SVEC endothelial cells, western blotting for PPAR-γ was performed using an antibody generated to a synthetic peptide corresponding to amino acid residues 284–298 of murine PPAR-γ; importantly, this antibody exhibits no significant homology to PPAR-α. This antibody recognized a PPAR-γ specific band at 55 kD by western blotting in SVEC cells (Fig. 1).

### TNF-α induced MAdCAM-1 protein expression is reduced by troglitazone, a PPAR-γ ligand

**Figure 2:**
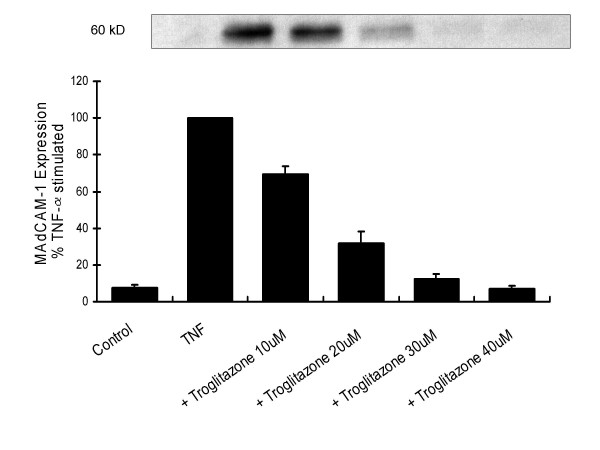
**TNF-α induced MAdCAM-1 protein expression is reduced by troglitazone, a PPAR-γ ligand**. TNF-α (1 ng/ml, 24 h) induced MAdCAM-1 expression was dose dependently blocked by the PPAR-γ ligand, troglitazone at concentrations between 10 to 40 μM (Fig. 2).

To examine the effect of PPAR-γ activation on endothelial cell inflammatory responses, SVEC endothelial cells were treated with TNF-α either with or without a pre- and co-treatment with troglitazone. TNF-α (1 ng/ml, 24 h) increased MAdCAM-1 expression to 13.4 times that of the untreated control level. This TNF-α induced MAdCAM-1 was dose dependently attenuated by pre- and co-treatment with troglitazone (i.e. from 69% to 7% of TNF-α stimulated levels) at concentrations of 10 to 40 μM (Fig. 2). At 40 μM troglitazone, the MAdCAM-1 expression (7% of TNF-α stimulated) was not significantly different from untreated controls. Troglitazone alone did not alter the expression of MAdCAM-1 (*data not shown*). Troglitazone was not overtly toxic, and did not affect cell protein content (i.e. protein content per well), or change the expression of either actin or vimentin, two cell structural proteins (measured by total protein staining of transferred western blots) (*data not shown*).

To examine the effect of troglitazone on other ECAMs expression stimulated by TNF-α surface expression assay was performed.

### TNF-α induced VCAM-1 protein expression is reduced by troglitazone, a PPAR-γ ligand

**Figure 3:**
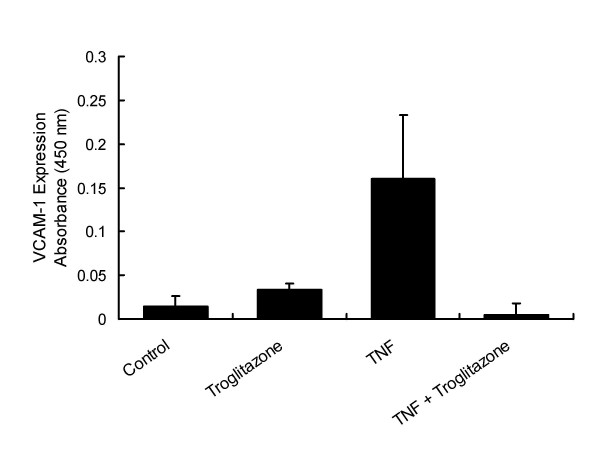
**TNF-α induced VCAM-1 protein expression is reduced by troglitazone, a PPAR-γ ligand**. TNF-α (1 ng/ml, 24 h) induced VCAM-1 expression was also blocked by the PPAR-γ ligand, troglitazone at 20 μM (Fig. 3). Alone, this compound had no effect on the expression of VCAM-1 (Fig. 3).

TNF-α (1 ng/ml, 24 h) enhanced VCAM-1 expression to 8.5 times that of untreated controls. This elevated expression was significantly reduced (to only 80% of the TNF-α stimulated level) by troglitazone at 20 μM (Fig. 3). Alone, this compound had no effect on the expression of VCAM-1 (Fig. 3).

### TNF-αinduced ICAM-1 protein expression is reduced by troglitazone, a PPAR-γ ligand

**Figure 4:**
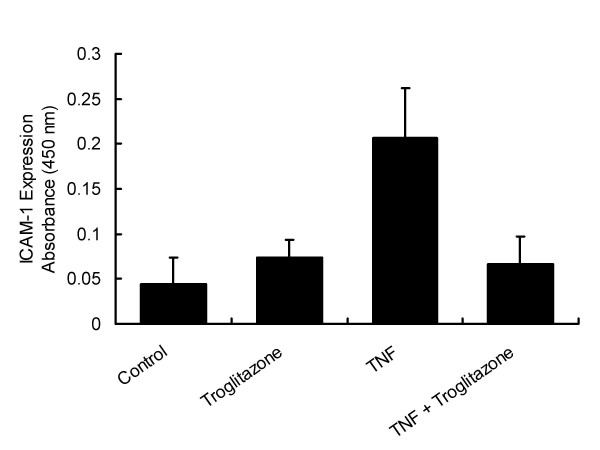
**TNF-α induced ICAM-1 protein expression is reduced by troglitazone, a PPAR-γ ligand**. TNF-α (1 ng/ml, 24 h) induced ICAM-1 expression was dose dependently blocked by the PPAR-γ ligand, troglitazone at 20 μM (Fig. 4). Alone, this compound had no effect on the expression of ICAM-1 (Fig. 4).

TNF-α (1 ng/ml, 24 h) increased ICAM-1 expression to a level 4.7 times greater than untreated control levels. This TNF-α enhanced ICAM-1 expression was strongly attenuated (to 32% of the TNF-α stimulated level) by troglitazone at 20 μM (Fig. 4). Again, troglitazone alone had no effect on the expression of ICAM-1 (Fig. 4).

### TNF-α induced E-selectin protein expression is reduced by troglitazone, a PPAR-γ ligand

**Figure 5:**
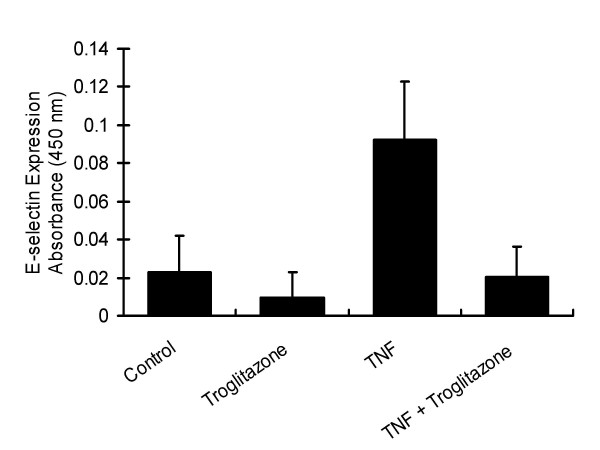
**TNF-α induced E-selectin protein expression is reduced by troglitazone, a PPAR-γ ligand**. TNF-α (1 ng/ml, 24 h) induced E-selectin expression was blocked by the PPAR-γ ligand, troglitazone at 20 μM (Fig. 5). Alone, this compound had no effect on the expression of E-selectin (Fig. 5).

TNF-α (1 ng/ml, 24 h) increased the expression of E-selectin 4 times over untreated control levels; this increase in E-selectin was also blocked by troglitazone (to 22% of the TNF-α stimulated level) at 20 μM (Fig. 5). Alone, troglitazone had no effect on E-selectin expression (Fig. 5).

### Adhesion of α4β7 expressing lymphocytes to TNF-α stimulated endothelium

**Figure 6:**
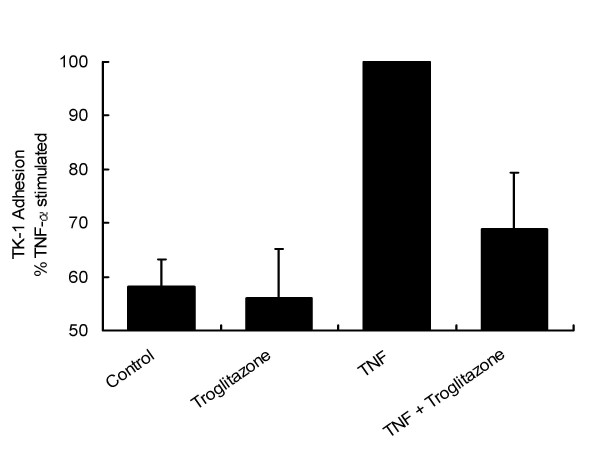
**Adhesion of α4β7 expressing lymphocytes (TK-1) to TNF-α stimulated endothelium**. TNF-α stimulation (24 h) significantly increased adhesion of TK-1 lymphocytes to monolayers of SVEC cells. Troglitazone (20 μM) significantly reduced TK-1 adhesion in response to TNF-α stimulation at 24 h (Fig. 6).

Having established that troglitazone exerts a significantly protective effect against TNF-α stimulated endothelial MAdCAM-1 induction, we examined the effects of troglitazone on the adhesion of α4β7 expressing mouse lymphocytes (using the cell line TK-1) to endothelial monolayers following TNF-α treatment. TNF-stimulation (24 h) significantly increased the adhesion of TK-1 lymphocytes to SVEC monolayers. Troglitazone (20 μM) significantly reduced TK-1 adhesion in response to TNF-α stimulation at 24 h (Fig. 6). Troglitazone did not modify the basal level of lymphocyte adhesion to the endothelium without TNF-α treatment.

### TNF-α induced phosphorylation of NF-kB p65 is prevented by troglitazone, a PPAR-γ ligand

**Figure 7:**
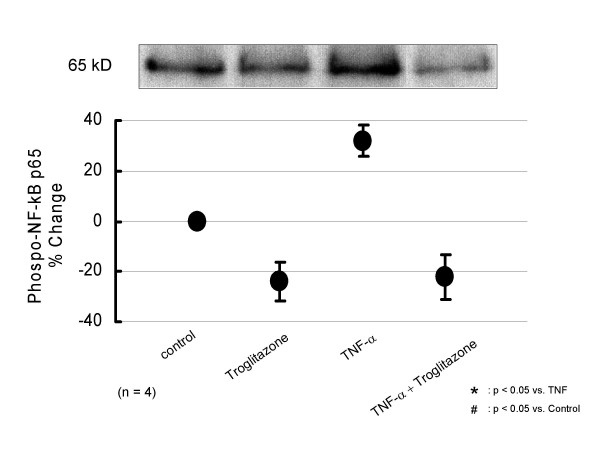
**TNF-α induced phosphorylation of NF-kB p65 is prevented by troglitazone, a PPAR-γ ligand**. TNF-α (1 ng/ml, 1 h) induced phosphorylation of NF-kB p65 was significantly attenuated by the PPAR-γ ligand, troglitazone (20 μM). Alone, troglitazone also attenuated phosphorylation of NF-kB p65.

Mechanistically, the expression of all of these adhesion molecules is known to depend on the activation of NF-kB following TNF-α stimulation. Therefore, we examined whether troglitazone-mediated protection against TNF-α induced MAdCAM-1 expression and lymphocyte adhesion were related to the activation and/or inhibition of NF-kB. In this study, phosphorylation of the p65 subunit of NF-kB was used as an index of NF-kB activation. TNF-α (1 ng/ml, 1 h) induced the phosphorylation of NF-kB p65; this activation was significantly reduced by the PPAR-γ ligand, troglitazone at 20 μM (Fig. 7). Alone, troglitazone significantly attenuated the phosphorylation of NF-kB p65 compared to untreated controls (Fig. 7).

## Discussion

MAdCAM-1 is a 60 KD endothelial cell surface molecule that is strongly expressed by mucosal endothelial cells, particularly following exposure of these cells to pro-inflammatory cytokines such as TNF-α. Expression of MAdCAM-1 has as well been reported in the brain, and in the heart, [23,47], and based on these findings, it has now been suggested that MAdCAM-1 might play roles in chronic inflammation of these organs as well.

With respect to inflammatory bowel disease, MAdCAM-1 appears to be necessary for lymphocyte homing to mucosa associated lymphoid tissue [3-5,50]. Since MAdCAM-1 is normally expressed within the gut microvasculature, and is dramatically increased during IBD, it has been suggested that increased MAdCAM-1 expression participates in the etiology of IBD through its ability to control homing of lymphocytes to the gut. This notion is supported by several observations that show that antibodies directed against either MAdCAM-1, or its lymphocyte ligand, the α4β7 integrin will significantly attenuate several indices of injury in experimental models of colitis [24,39].

TNF-α is thought to be perhaps the most important cytokine responsible for driving the onset and progression of IBD. Because of this primary role of TNF-α in IBD, anti-TNF-α antibody therapy has been successfully used in IBD to reduce both colonic injury and expression of ECAMs in IBD [1]. While TNF-α significantly increased the expression of MAdCAM-1, it also increases the expression of intercellular cell adhesion molecule-1 (ICAM-1), vascular cell adhesion molecule-1 (VCAM-1), E-selectin, P-selectin [15,37]. It should be noted that in colitis, all of these adhesion molecules are elevated in the colon [25], and likely contribute to the development of chronic gut injury.

This is the first study to demonstrate that a PPAR-γ ligand, troglitazone, can significantly reduce the expression of MAdCAM-1, an endothelial cell adhesion molecule which is closely linked to chronic gut inflammation. Troglitazone significantly reduced TNF-α induced expression of several other ECAMs as well [6,21,30,32,38,41,57], and decreased the adhesion of α4β7-expressing lymphocytes (TK-1) to TNF-α stimulated endothelium. Since at least 50% of the adhesion of these lymphocytes to TNF-stimulated endothelium is MAdCAM-1-dependent [35], our results suggest that MAdCAM-1 mediates most of the stimulated adhesion, with more minor contributions from other ECAMs. The results strongly support a novel therapeutic action of PPAR-γ activators like troglitazone which might explain their beneficial effects PPAR-γ agonists in murine models of colitis [12,51] and in human ulcerative colitis [27].

NF-kB is a member of the Rel family of dimeric transcription factor complexes key transcription factor that modulates expression of MAdCAM-1 in inflammation, and is governs the expression of several other endothelial adhesion molecules in response to cytokines [9,22,33,35,40,52]. Prior to cytokine stimulation, NF-kB is restricted to the cytosol as an inactive complex with its inhibitor, Ik-B. Upon activation by cytokines, Ik-B is phosphorylated, dissociates from the NF-kB, and is subsequently ubiquitinated and degraded. This allows active NF-kB to enter the nucleus and bind kB consensus regulatory elements in the promoters of the genes for several ECAM (ICAM-1, VCAM-1, E-selectin, and MAdCAM-1) [9,33-36,54]. NF-kB can be activated through several kinases including IkB kinases (IKKs). IKKs phosphorylate IkB, but also phosphorylate the p65 NF-kB subunit on Serine-536 as part of the activation of the NF-kB complex. Phosphorylation of the p65 subunit is an important step in the activation of the NF-kB complex which permits this complex to enter the nucleus and activate NF-kB dependent genes [45]. Consequently, phosphorylation of p65 has been proposed as a simple index of NF-kB activation [45], although it may not be as sensitive as the electrophoretic mobility shift assay (EMSA).

PPAR-γ ligands like troglitazone apparently suppress activation of NF-kB, and in our hands reduce both basal and TNF-stimulated NF-kB p65 phosphorylation (figure 7). Consequently, glitazones like troglitazone should reduce the expression of both cytokines and ECAMs driven by NF-kB [6,41,57]. Interestingly, under control culture conditions, we observe basal phosphorylation of p65, suggesting that normally, p65 is at least partially activated. This may be related to the role of basal NF-kB activity in maintaining cell survival and blocking apoptosis [8,42]. Since excessive inhibition of NF-kB can propel cells into apoptosis, agents like troglitazone, (which may inhibit NF-kB) could have a limited therapeutic window, and should be administered cautiously. However, under the conditions used in our study, (10–40 μM) we did not see evidence for the loss of cell viability assessed by trypan blue staining; all cells remained >99% viable by this method. In addition, the concentration of troglitazone in our study (10–40 μM) is near the therapeutic level since the physiological levels of glitazones, like troglitazone are 5–20 μM, with an average dose of 15 μM [28].

PPAR-γ ligands like glitazones should not only attenuate MAdCAM-1, but also diminish the expression of other ECAMs like ICAM-1, VCAM-1 and E-selectin. This reflects a decrease in the synthesis of some, but not all proteins, since densitometry of troglitazone treated monolayers shows no difference in total protein content between wells following troglitazone, but western blotting or surface expression assay find a significant decrease in the expression of ECAMs.

While studies with glitazones in endothelial models for the most part demonstrate an *inhibition *of ECAMs such as ICAM-1 in response to TNF-α [10,38], Chen et al. [6,7] have reported that the ECV304 cell line showed an increase in the expression of ICAM-1 in response to troglitazone. This stands in sharp contrast to our current study. However, since The ECV304 cell line has been mistakenly designated as '*endothelial*' in many cases, (and is actually a bladder carcinoma in many instances) [56], those results may be called into question.

It is also possible that PPAR-γ activators might well affect inflammation through NF-kB *independent *pathways. Some described PPAR-γ ligands, like the cyclopentanone prostanoids, exert anti-inflammatory effects through a PPAR-γ-independent pathways [43] specifically the inhibition of the IKK beta subunit which would suppress NF-kB activation. Further, troglitazone also activates ERK1/2 [20] and blocks c-fos synthesis [6] which could also modulate these effects. Therefore, we cannot completely exclude the possibility that troglitazone similarly protects in these models through direct inhibition of IKK, as well as indirect blockade through PPAR-γ.

Since high levels of PPAR-γ protein are expressed within the colon, [13] it is possible that agonists for this pathway could have an important role in the regulation of normal colon functioning and disease progress.

## Conclusions

Our results indicate that troglitazone and other glitazones may provide an effective means of treating forms of chronic inflammation including inflammatory bowel disease through their ability to interfere with steps in the activation of NF-kB, effectively blocking the expression of adhesion molecules like MAdCAM-1 which increase infiltration of tissues by leukocytes.

## Methods

### Reagents

Recombinant mouse TNF-α was purchased from ENDOGEN (Stoughton, MA), and troglitazone was provided as a generous donation from Sankyo corp., Japan.

### Cell culture

The SVEC4-10 line is an endothelial cell line derived by SV40 (strain 4A) transformation of murine small vessel endothelial cells, originally isolated from the axillary lymph node vessels of an adult male C3H/Hej mouse [4,5]. These cell types were all maintained in Dulbecco's modified Eagle's medium (DMEM) with 10% fetal calf serum with 1% antibiotic/ antimycotic. Cells were seeded into 24-well tissue culture plates at approximately 20,000 cells/cm^2^, and cultures were used immediately upon reaching confluence (usually 3–4 days after seeding).

### Lymphocytes

The mouse CD8^+ ^T cell lymphoma TK-1 cells (that constitutively expresses the α4β7 integrin [44] were obtained as a generous gift from Dr. Eugene Butcher (Stanford University, CA). These cells were cultured in RPMI-1640 medium supplemented with 10% FCS and 0.05 mM 2-mercaptoethanol (minus antibiotic/ antimycotic).

### PPAR-γ expression on SVEC endothelial cell – Western analysis of cell lysates

Cell lysates were electrophoretically separated on 7.5% SDS- PAGE gels, transferred to nitrocellulose, blocked and incubated in primary anti-PPAR-γ synthetic peptide (Affinity Bioreagents Inc., Golden, CO) at a 1: 500 dilution overnight (4°C). Membranes were washed 2× with wash buffer. Secondary goat anti-rabbit horseradish peroxidase conjugated secondary antibody (Sigma) was added at a 1:2,000 dilution for 1 h. Membranes were washed 3 times and developed using the ECL detection system (Amersham, La Jolla, CA).

### Western analysis of cell lysates

Monolayers were either pretreated (1 h) with blockers, and then incubated with cytokines (24 h), or treated without test agents and then treated with cytokines (24 h). All cell samples were harvested at 24 hours. Equal quantities of protein (75 μg) from each sample were electrophoretically separated on 7.5% SDS- PAGE gels. Gels were transferred to nitrocellulose membranes (Sigma) and blocked with 5% milk powder in PBS at 4°C (overnight). These membranes were washed twice for 10 min with wash buffer (0.1% milk powder in PBS). Primary rat anti-mouse MAdCAM-1 mAb (MECA 367; generous gift from Dr. Sharon Evans, RPMI, NY) was added at a concentration of 10 μg/ml and incubated at room temperature for 2 h. In p65 NF-kB phosphorylation studies, membranes were incubated with anti-phospho p65 antibody (Cell Signaling Technology, Beverly, MA) diluted 1:1000 overnight (4°C). These membranes were washed twice with wash buffer. Secondary goat anti-rat horseradish peroxidase conjugated secondary antibody (Sigma) was added at a 1:2000 dilution for 2 h for MAdCAM-1, while goat anti-rabbit antibody was used for detecting phospho-p65 NF-kB. Lastly, membranes were washed 3 times and developed using the enhanced chemiluminescence (ECL) detection system. The density of MAdCAM-1 staining was measured by scanning the 60 KD band, using a HP ScanJet™ flatbed scanner. Images were analyzed for density using Image Pro Plus™ image analysis software (Media Cybernetics, Silver Springs, MD). The data are expressed as a percentage of TNF-α-induced level of density.

### Endothelial cell surface adhesion molecule expression assay

Surface expressions of ECAMs were measured using the method of Khan et al. [26]. SVEC monolayers were grown in 48-well plates as described and were pretreated (1 h) with troglitazone and of 1 μg/ml in HBSS/PBS + 5% FCS at 37°C for 30 min. Monolayers were then washed twice with 0.5 ml HBSS/PBS, and incubated with horseradish peroxidase conjugated rabbit anti-rat IgG (1:2,000 diluted, Sigma) in HBSS/PBS + 5% FCS at 37°C for 30 min. Monolayers we then co-treated with TNF-α (1 ng/ml) at 37°C in medium for 24 h. The cells were washed three times with 0.5 ml HBSS/PBS [1:1] at 24 hours, and monolayers incubated with anti-mouse VCAM-1, anti-ICAM-1 or anti-E-selectin. All antibodies were added to cultures after treatment at a concentration re washed four times with 0.5 ml HBSS/PBS followed by incubation with 0.25 ml of 0.003% hydrogen peroxide + 0.1 mg/ml 3, 3', 5, 5'-tetramethlbenzidine (Sigma) at 37°C for 60 min in the dark. The color reaction was stopped by adding 75 μl of 8 N H_2_SO_4_, and the samples were transferred to 96-well plates. Plates were read on a Titertek MCC340 plate reader (Titertek Instruments, Inc., Huntsville, AL) at 450 nm. Blanking (i.e. background) was performed on monolayers stained only with second antibody and reacted as above.

### TK-1 lymphocyte adhesion assay

Briefly, TK-1 cells were suspended in culture medium and fluorescence labeled by incubating TK-1 cells at 2 × 10^6 ^cells/ml with 0.02 mg fluorescein diacetate (FDA) (Sigma) at 37°C for 30 min. The cells were then washed twice with ice-cold HBSS, spun at 250 g for 5 min to remove unincorporated fluorescence and suspended in HBSS. The TK-1 lymphocyte cell line used in this assay expresses high levels of the α4β7 integrin, [35,44] which can interact with multiple ligands including mucosal addressin-1 (MAdCAM-1), as well as VCAM-1, L-selectin and fibronectin [18]. In this system, TNF stimulated TK-1 adhesion to SVEC4-10 endothelial cells is at least 50% MAdCAM-1 dependent [35]. SVEC monolayers were grown in 48-well plates as described, and to activate endothelium, the monolayers were incubated with TNF-α (1 ng/ml) for 24 h. Cytokine treated endothelial cells were washed three times with media. Labeled TK-1 cells were then added to the endothelium at a 5:1 lymphocyte to endothelial cell ratio [31] and allowed to bind for 30 min under static conditions. At the end of the incubation period, the supernatant was removed and the Monolayers were washed twice with HBSS. Plates were read on a Fluorescent Ascent (Lab systems, Helsinki, Finland) set for excitation at 485 nm, and emission at 515 nm. Blank wells (0% TK-1 adhesion) were run as controls that did not contain labeled TK-1 cells; 100% adhesion was measured on monolayers where TK-1 cells were not removed from the supernatant.

## Abbreviations

PPAR-γ – peroxisome proliferative activated receptor gamma

MAdCAM-1 – Mucosal Addressin Cell Adhesion Molecule-1

VCAM-1 – Vascular Cell Adhesion Molecule-1

E-selectin – Endothelial Selectin

ICAM-1 – Intercellular Adhesion Molecule-1

IBD – Inflammatory Bowel Disease

TNF-α – Tumor Necrosis Factor-α

## Authors' contributions

Dr. Sasaki accomplished the studies described in this manuscript, all authors contributed equally in the design, interpretation and execution of this article.
